# *Melissa officinalis*: Composition, Pharmacological Effects and Derived Release Systems—A Review

**DOI:** 10.3390/ijms23073591

**Published:** 2022-03-25

**Authors:** Gabriela Petrisor, Ludmila Motelica, Luminita Narcisa Craciun, Ovidiu Cristian Oprea, Denisa Ficai, Anton Ficai

**Affiliations:** 1 Science and Engineering of Oxide Materials and Nanomaterials, Faculty of Applied Chemistry and Materials Science, University Politehnica of Bucharest, 011061 Bucharest, Romania; gabriela.petrisor06@yahoo.com (G.P.); motelica_ludmila@yahoo.com (L.M.); 2 National Research Center for Food Safety, University Politehnica of Bucharest, 060042 Bucharest, Romania; ovidiu73@yahoo.com (O.C.O.); denisaficai@yahoo.ro (D.F.); 3 National Center for Micro and Nanomaterials, University Politehnica of Bucharest, 060042 Bucharest, Romania; 4 Department of Inorganic Chemistry, Physical Chemistry and Electrochemistry, Faculty of Applied Chemistry and Materials Science, University Politehnica of Bucharest, 011061 Bucharest, Romania; luminita.craciun@upb.ro; 5 Academy of Romanian Scientists, 050044 Bucharest, Romania

**Keywords:** *Melissa officinalis*, essential oil, polyphenolic compounds, pharmacological effects, controlled release system

## Abstract

*Melissa officinalis* is a medicinal plant rich in biologically active compounds which is used worldwide for its therapeutic effects. Chemical studies on its composition have shown that it contains mainly flavonoids, terpenoids, phenolic acids, tannins, and essential oil. The main active constituents of *Melissa officinalis* are volatile compounds (geranial, neral, citronellal and geraniol), triterpenes (ursolic acid and oleanolic acid), phenolic acids (rosmarinic acid, caffeic acid and chlorogenic acid), and flavonoids (quercetin, rhamnocitrin, and luteolin). According to the biological studies, the essential oil and extracts of *Melissa officinalis* have active compounds that determine many pharmacological effects with potential medical uses. A new field of research has led to the development of controlled release systems with active substances from plants. Therefore, the essential oil or extract of *Melissa officinalis* has become a major target to be incorporated into various controlled release systems which allow a sustained delivery.

## 1. Introduction

Plants are the oldest health remedy, and have been known to people since antiquity. Over the centuries, various cultural groups have developed traditional herbal medical systems to improve health. From the beginning of the 19th century, the isolation of active compounds from plants began; due to the rapid developments in the field of chemistry, from the beginning of the 20th century the production of synthetic compounds increased [1]. Even though the use of synthetic compounds has grown in the drug industry, most developing countries continue to use drugs made from natural compounds [2]. Medicinal plants have a variety of biological properties, which means that they play an important role in preventing and treating various diseases [3]. These plants are a rich source of biological active agents, and can represent raw materials that can be used to develop new, semi-synthetic drugs. 

The active substances are found in different parts of the plant, and can be extracted from different types of seeds, roots, leaves, fruits, skin, flowers, or even the whole plant. The active compounds extracted from most medicinal plants have direct or indirect therapeutic effects.

**Figure 1:**
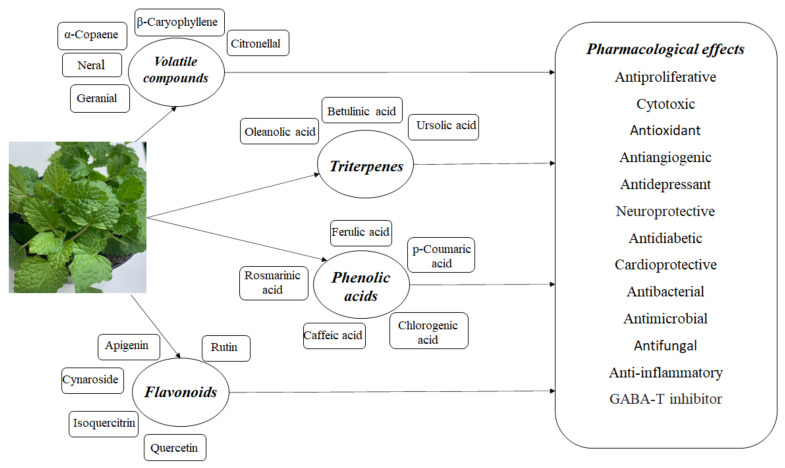
The composition of *Melissa officinalis* and its pharmacological effects.

*Melissa officinalis* L. is a medicinal plant used in traditional medicine around the world. It is an aromatic perennial plant; it commonly grows in the Mediterranean region and Western Asia, being intensively cultivated in Europe [4,5] and, due to its chemical composition and numerous pharmacological effects, it is intensively studied. This plant is also called lemon balm, honey balm, or balm mint; it is an edible medicinal herb belonging to the mint family Lamiaceae and the subfamily Nepetoideae [5]. It is a plant that lives at least three years; it is bushy and upright, reaching a height between 60 and 100 cm [6]. The soft, hairy leaves are 2 to 8 cm long; they are dark green and heart shaped. The leaf surface is coarse and deeply veined, the leaf edge is scalloped or toothed [7], and it is rich in biological active agents; thus, the extracts highlight specific properties (Figure 1). *Melissa officinalis* has a hairy root system, which makes the plant more adaptable to different environmental conditions, but the upper parts of the plant die out in early winter and reappear in early spring [7]. It is one of the easiest herbs to grow, and spreads so readily that some gardeners consider it a weed [8].

This paper provides an overview of the phytochemical composition of *Melissa officinalis*, a plant which is rich in volatile compounds and polyphenolic compounds. Research on the determination of its composition and biological activities has led to the discovery of numerous pharmacological agents with different activities, such as antioxidant, antimicrobial, and cytotoxic activities, and many others. Given the development of nanotechnology and the desire to create new systems of controlled release, we highlighted systems that contain bioactive compounds such as essential oil or *Melissa officinalis* extract, and also systems with pure active substances. The diversity of the materials and their dimensions has led to the formation of controlled release systems with many biological effects. This review was especially focused on highlighting studies carried out on different controlled release systems that contain—as bioactive component—essential oil or extract, as well as the pure substances available from *Melissa officinalis*. 

## 2. Phytochemical Composition

Chemical studies on the composition of the *Melissa officinalis* have shown that it contains mainly flavonoids, terpenoids, phenolic acids, tannins and essential oil [9]. The main active constituents of *Melissa officinalis* are volatile compounds (geranial, neral, citronellal and geraniol), triterpenes (ursolic acid and oleanolic acid), phenolic compounds (rosmarinic acid, caffeic acid, and protocatechuic acid) and flavonoids (quercetin, rhamnocitrin, luteolin) [8,10]. Essential oil is usually considered to be the responsible therapeutic principle for most biological activities, but polyphenols are also involved.

*Melissa officinalis* essential oil—obtained from the fresh or dried flower, leaf, and branches of this plant by water steam distillation or chemical extraction—is characterised by a fresh lemon odor and light yellow color [11].

### 2.1. Volatile Compounds

The volatile oil extracted from the leaves of *Melissa officinalis* is important due to its pharmacological effects, and is obtained in small quantities, unlike other plants in the Lamiaceae family. The major and minor components of the essential oil of the dried leaves of *Melissa officinalis* are volatile compounds that are found in different concentrations (Table 1).

Kowalski et al. [19] reported a 0.17% essential oil content, which was very low, unlike plants in the same family. The study by Seidler-Łożykowska et al. [16] showed that the essential oil content ranged from 0.08 to 0.20% due to fluctuations in weather conditions during the research years. For the variability of the essential oil content and its composition, Kittler et al. [20] studied a set of 28 accessions of lemon balm. They obtained an essential oil content that varied between 0.01 and 0.72%, and found out—based on statistical analysis on the composition of the essential oil—that there are two chemotypes of essential oil: chemotype citral and chemotype germacrene D. As it is known from the literature, there are two subspecies of the plant *Melissa officinalis*: *officinalis* and *altissima*. The difference between the two subspecies is given by the composition of the essential oil, such that the subspecies *officinalis* contains major amounts of citral and/or neral, but the subspecies *altissima* contains only traces. Basta et al. [21] reported, in a study on the composition of the essential oil of *Melissa officinalis* in Greece, the lack of the major constituents citral and citronellal. Souihi et al. [14] reported, in a comparative study between *Melissa officinalis* from Tunisia (from two different cities), Germany, and France, the lack of citral but the major presence of germacrene D.

The citrus-like aroma of *Melissa officinalis* is due to the presence of citral isomers, as well as lesser amounts of citronellal and geranyl acetate [10]. Citral is an acyclic monoterpene aldehyde which consists of a racemic mixture of two isomers: geranial (trans-citral or citral A) and neral (cis-citral or citral B); it is the major compound in the essential oil of *Melissa officinalis*, subspecies *officinalis*. In a study carried out on plants from the Lamiaceae family, Kowalski et al. [19] reported the highest content of geranial (23.3%), followed by neral (16.0%) and caryophyllene oxide (15.8%) in *Melissa officinalis*.

Nurzynska-Wierdak et al. [13] investigated changes in the chemical composition of the dried oil of dried leaves of *Melissa officinalis*, from Poland, in the first and second year of growth. They obtained, by hydrodistillation from air-dried leaves, 0.3% essential oil; by analysis with GC-MS and GC-FID they highlighted 106 compounds, of which the predominant components were geranial (45.2% and 45.1%) and neral (32.8% and 33.8%). Barakat et al. [22] studied the chemical composition of Jordanian *Melissa officinalis* essential oil, and found that it differed significantly from the composition of *Melissa officinalis* essential oil from other countries.

In the comparative study performed by Souihi et al. [14], it was found that the essential oil of the French population showed lower levels of geranial (7.12%) and neral (4.29%) than that of the German population, which contains 39.31% geranial, 27.71% neral, and 12.23% β-caryophyllene. Nouri et al. [12] conducted a study on the composition of essential oil and rosmarinic acid in *Melissa officinalis* grown in southern Iran, and obtained a total yield of 0.37% (*v*/*w*), rich in geranial and neral; compared to other research, they discovered a high content of rosmarinic acid.

In another study, the essential oil obtained from leaves of *Melissa officinalis* growing in Algeria was investigated for its chemical composition and in vitro antimicrobial activity [15]. In the essential oil—obtained by hydrodistillation and analyzed by GC-MS and GC-FID—sixty-three compounds were identified, representing 94.10% of the total oil, and the yield was 0.34%. The major component was geranial (44.20%), and other predominant components were neral (30.20%) and citronellal (6.30%). 

Seidler-Łożykowska et al. [16] specified in their study that the main components, neral and geranial, had higher concentrations under higher insolation. The five most active components identified in the essential oils extracted from plant materials were geranial, neral, citronellal, caryophyllene oxide and β-caryophyllene. In this study, the following results were obtained: the amount of geranial ranged from 6.22% to 21.49%, the level of neral ranged from 4.28% to 15.08%, the citronellal content ranged from 2.80% to 19.74%, the level of caryophyllene oxide varied from 20.86% to 41.72%, and high levels of β-caryophyllene were observed, ranging from 12.08% to 29.14%.

Based on the results obtained by Said-Al Ahl et al. [18], it was found that the harvesting time influences the essential oil content and quality of *Melissa officinalis*. The highest yield of essential oils was obtained when harvested in September, August and October, and the lowest was obtained when harvested in January, February and March. According to these results, it is recommended to harvest in warmer months in order to obtain a high content of essential oil. The geranial (23.8–51.2%) and neral (20.1–35.0%) content of the essential oil extracted from the fresh leaves of *Melissa officinalis* was highlighted for different months of harvest.

The chemical composition of the essential oil has a high variability, and depends on the origin of the plant, the climatic difference, the geographical conditions, the cultivation conditions, the harvesting time, and the techniques applied for the extractions [10,13].

### 2.2. Triterpenes

Triterpenes are non-volatile components extracted from plants, and are one of the largest classes of natural plant products, with more than twenty thousand different triterpenes (Table 2) [23,24]. Some triterpenes contain a different number of sulfate groups bound to sugar or glucones [10].

The most common triterpenes in *Melissa officinalis* extracts are ursolic acid and oleanolic acid. In the studies of Ghiulai et al. [4] and Ibarra et al. [27], ursolic acid and oleanolic acid were extracted together with polyphenolic compounds in order to show the biological properties of the plant. Using different extraction conditions, different extracts can be obtained with different compositions, and thus different activities. The first extraction was obtained by maceration for 9 days in 70% ethanol, the second was obtained by maceration in 96% ethanol for 24 h under continuous stirring, and the third was extracted by sonication in 80% methanol for one hour. In the three extractions, the largest amount of ursolic acid was obtained, followed by oleanolic acid and small amounts of betulinic acid. Comparing the values of the obtained concentrations, the extraction in methanol with sonication is the most favorable, obtaining the best results: ursolic acid (11,234.97 μg/g), oleanolic acid (6151.67 μg/g) and betulinic acid (169.88 μg/g).

There are very few studies on the non-volatile components of *Melissa officinalis*. Mencherini et al. [24,26] found five disulfated ursene triterpenes and an ursenic glycoside from an extract of dried stems and leaves, and two sulfated triterpenes in an extract of fresh leaves and stems.

In a recent study, Abdel-Naime et al. [25] identified three ursene triterpene glycosides and a known 23-sulfate triterpenoid glycoside ester of niga-ichigoside F1, which was isolated before by Mencherini et al. [24]. The three ursene triterpenes glycoside discovered were Melissioside A-C.

Triterpenes are found in *Melissa officinalis* in high concentrations, along with numerous polyphenolic compounds.

### 2.3. Polyphenolic Compounds

Polyphenolic compounds are a group of secondary metabolites which includes flavonoids (e.g., anthocyanins, flavones, isoflavones) and phenolic acids, which have many biological properties (Table 3) [28].

Phenolic acids compose a large group of natural compounds, which exhibit a wide range of biological activities. Flavonoids are a class of polyphenolic compounds, classified according to their chemical structures into flavonols, flavones, flavanones, isoflavones, catechins, anthocyanidins and chalcones [31]. Phenolic acids and flavonoids contain chemical structural elements that are responsible for the antioxidant process, and their antioxidant activities have been well established biochemically. Phenolic acids are important bioactive constituents of *Melissa officinalis*, among which are rosmarinic, caffeic, chlorogenic and ferulic acids. Following the phenolic profiles of the three types of extracts from the study performed by Ghiulai et al. [4], it was observed that rosmarinic acid was obtained in the highest amount (86,637.60 μg/g). The extraction with methanol, by sonication, favored the extraction of all of the phenolic compounds sought; thus, it can be observed that, depending on the extraction technique, some compounds can be in high concentrations and others cannot be detected.

Barros et al. [28] carried out a comparative study on the phenolic profiles of commercial samples and samples grown in the garden, prepared by infusion. In the samples were identified rosmarinic acid, as the main component, and derivatives of caffeic acid (trimers: lithospermic acid, salvianolic acid A, C and F and yunnaneic acid F; tetramers: salvianolic acid B and sagerinic acid). Despite the similar profile observed in all of the studied lemon balm samples, the quantities found of each compound were different. Cultivated and in vitro cultured samples presented the lowest quantities of phenolic compounds (59.59 and 30.21 mg/g infusion), whereas commercial samples showed the highest contents (109.24 mg/g for a tea bag and 101.03 mg/g for a granulate sample).

Through extraction techniques—enzyme-assisted extraction and pressurized liquid extraction—Miron et al. [32] obtained extracts from *Melissa officinalis* which were very rich in caffeic acid and rosmarinic acid derivatives, some of which were identified for the first time in this plant, such as: salvianolic acid H/I, salvianolic acid E, salvianolic acid L and the salvianolic acid L isomer.

Gallic acid is one of the biologically active phenolic acids that was reported in the study of Perreira et al. [33] as an important phenolic acid for the antioxidant and anticholinesterase activity of *Melissa officinalis*.

In a study of seven medicinal plants (from five plant families) grown in Slovakia, Gayibov et al. [30] investigated the content of polyphenols and the content of flavonoids, as well as the antioxidant activity and antimicrobial activity. This study included three plants of the Lamiaceae family: *Melissa officinalis*, *Salvia officinalis* and *Thymus pannonicus*. The polyphenolic content obtained is quite similar (20.90 ± 1.06, 20.01 ± 0.71 and 23.98 ± 1.37 mg gallic acid equivalents/g sample), but the flavonoid content varied lightly (11.56 ± 0.15, 14.35 ± 0.49 and 19.35 ± 1.22 mg quercetin/g of sample). In this study, it was observed that *Melissa officinalis* contained the highest content of rosmarinic acid (6914.1 ± 779), but some of the compounds which followed were missing.

Spiridon et al. [34] conducted a comparative study on the polyphenol content of some important medicinal plants in Romania, originating in the southeastern region, such as oregano (*Origanum vulgare*), lavender (*Lavandula angustifolia*) and lemon balm (*Melissa officinalis*). In this study, the extraction yield for plants, calculated on the dry weight of the raw material, was as follows: 7.89% for lavender, 11.38% for oregano, and 11.93% for lemon balm. In lemon balm, the total content of phenolic compounds (54.9± 2.14 mg gallic acid equivalents/g) and flavonoids (25.8 ± 6.26 mg rosmarinic acid/g) was identified to be higher than in lavender but lower than in oregano.

In another study conducted on *Melissa officinalis* grown in Romania, Fierascu et al. [29] highlighted the phytochemical composition of the ethanolic extract from dried leaves, which was obtained by the accelerated extraction of the solvent. In this study, high values were obtained for chlorogenic acid (72.529 ± 0.24 mg/kg), ferulic acid (45.489 ± 0.15 mg/kg), quercetin (153.465 ± 0.32 mg/kg) and rutin (1462.997 ± 1.24 mg/kg).

*Melissa officinalis* is a plant which is rich in polyphenolic compounds, but depending on the extraction method and the solvent used, different compositions of the extracts can be obtained; this influences the biological activities strongly.

### 2.4. Other Components

As presented above, *Melissa officinalis* is a source of active biocompounds such as volatile compounds, triterpenes and polyphenolic compounds, but, in addition to these, it also contains other important biological active agents. Ashori et al. [35] conducted a study on the chemical composition of the stalk of *Melissa officinalis*. The content of extractive agents, lignin, polysaccharides and ash was determined, with the observation that the results showed a high content of alpha-cellulose and a low content of lignin. Komes et al. [36] reported the content of tannins, phenolic acids and flavonoids in non-hydrolyzed and hydrolyzed extracts from various plants, including *Melissa officinalis*. Dias et al. [37] performed a comparative study between two commercial samples, an in vitro culture sample and a normal culture sample. After the analysis of all of the samples, they observed the highest levels of proteins (8 g/100 g dw) and ash (12 g/100 g dw) in the in vitro cultured lemon balm content; the highest levels of carbohydrates were found in the granulate commercial sample (85 g/100 g dw), and a bag of commercial lemon balm had the highest energetic value (377 kcal/100 g dw) due to its higher fat content (3 g/100 g dw).

Overall, *Melissa officinalis* has a complex chemical composition, with many active biocompounds, which differ depending on the way in which the extraction is performed and the part of the plant that is subjected to extraction.

## 3. Pharmacological Studies

According to many biological studies, plant extracts and volatile oils are known for many beneficial activities for the human body. *Melissa officinalis* is considered to be a medicinal plant due to the numerous pharmacological effects associated with its chemical composition (Table 4).

Some of the pharmacological activities may be connected with the polyphenolic compounds occurring in *Melissa officinalis*, which include phenolic acids and flavonoids [45,58]. Most studies have focused on *Melissa officinalis* leaf extracts, obtaining phenolic profiles correlated with antiproliferative [39], antiangiogenic [4], antiviral [55,59], antioxidant [41,42], anti-anxiety [45], antidepressant [45], anti-Alzheimer [53], neuroprotective [46], cardioprotective [43], antifungal [56,57] and antibacterial [42] effects. Moaca et al. [38] performed a comparative study between extractions from stems and leaves in order to evaluate the antioxidant activity, the total phenolic contents, and the cytotoxic and antiproliferative effects. In this study, a good antioxidant activity was observed to be correlated with the high total polyphenol content of the leaf ethanolic extract (32.76 mg gallic acid equivalents/g) as opposed to the seed ethanolic extract (8.4 mg gallic acid equivalents/g). The extracts obtained showed a different profile of cytotoxic effects on breast cancer cells, MDA-MB-231, but with significant antitumor activity for future in vitro studies. Ghiulai et al. [4] investigated the potential for angioprevention and chemoprevention in breast cancer from various extracts of *Melissa officinalis*. The antioxidant activity and in vitro effect on cell viability were evaluated on two breast cancer cell lines, MCF7 and MDA-MB-231. Based on the evaluation in ovo, using the chorioallantoic membrane test, it was found that 96% ethanolic extract is the strongest chemopreventive agent.

The ethanolic extract of *Melissa officinalis* showed an antiproliferative effect on the human colon cancer cell line (HCT-116) [39], as well as a strong antitumor effect on the human tumor cell lines MCF-7, AGS and NCI-H460 [40].

Many studies have shown the good antioxidant activity of *Melissa officinalis* extracts, which is an important step in the identification of the various beneficial effects on the human body. Perreira et al. [41] suggested that this plant could be used as a potential agent for the prevention of various neurological diseases associated with oxidative damage, reporting the best antioxidant activity and the highest content of reducing agents compared to *Matricaria recutita* and *Cymbopogon citratus*.

In vitro studies have shown potential pharmacological effects of Melissa officinalis extracts on specific cells or tissues, but in vivo animal studies are needed in order to confirm these effects.

The results of in vivo studies in mice have shown that *Melissa officinalis* extract can be considered a protective agent in various neurological diseases associated with ischemic brain injury [46], and can inhibit anxiety and depression [45].

In vivo studies have shows the pharmacological effects of doses of *Melissa officinalis* extracts in animals. In order to develop new drugs, it is necessary to move forward from the equivalent dose for animals to the equivalent dose for humans. There are different calculation models for the estimation of the equivalent dose in humans based on existing studies. The estimation of the human equivalent dose is an important step in the development of a new herbal medicine in order to further evaluate the toxic or safety aspects. In order to facilitate the interpretation of the results of in vivo studies, we converted animal doses to human equivalent doses (*HED*) with the following formula [60]:

$$HED\;\left[\frac{mg}{kg}]=Animal\;dose\;[\frac{mg}{kg}\right]*{\left(\frac{Animal\;weight\;\left[kg\right]}{Human\;weight\;\left[kg\right]}\right)}^{0.33}$$

Based on the estimated human equivalent doses, it can be concluded that antihyperglycemic activity is assured with 0.38–1.5 mg/human/day, while cardioprotective activity can be assured with 253.8–1014 mg/human/day, and anxiolytic activity can be assured with 234–703 mg/human/day; these values are comparable to the drugs available on the market [61,62,63]. In the case of neuroprotection, the estimated human equivalent dose is 501–4000 mg/human/day, close to the value for phenobarbital, which is administered at 40 mg/kg [64]. Analgesic activity can be assured with only 52–207 mg/human/day, which is many times lower than the daily dose for other available drugs [65]; however, for hypnosis a much higher estimated dose is necessary, such as 888–3554 mg/human/day [66,67,68]. In all cases, the estimation of the daily dose was carried out for an average body weight of 60 kg. However, it must be taken into account that biochemical processes vary between species, and that clinical trials are needed in order to ensure that a new drug is effective and safe.

Table 5 presents some of the most important components of *Melissa officinalis* extracts, along with their documented antimicrobial, antioxidant or anti-inflamatory activities. The biological profile of the essential oil or extracts from *Melissa officinalis* is the direct result of these compounds’ presence. One natural successful strategy to enhance a property, be it antioxidant or antimicrobial activity, is to combine multiple compounds with the desired property. The synergy will allow the manifestation of a higher level of antibacterial activity, for example, while keeping the concentration low. This is also a successful strategy to combat microbial acquired resistance.

The synergic activity was well documented in the literature for several classes of drugs/substances, with some of these effects being reviewed already for antibiotics and plant extracts/essential oils even in multidrug-resistant staphylococci [103].

*Melissa officinalis* and other plant extracts (*Iberis amara*, *Silybum marianum* and a mixture of *Angelica archangelica* and *Carum carvi*) were evaluated in association with STW5, a well-known fixed herbal multicomponent preparation recommended, at least, by the German treatment guideline for some gastrointestinal diseases under non-inflammatory and inflammatory conditions. It was found that only *Melissa officinalis* plant extract manifested a strong synergic effect when intestinal smooth muscle cells were considered [104]. The oral administration of a *Melissa officinalis* infusion can be also beneficial for the radiology staff, as the oxidative stress and DNA damage are reduced [105]. At present, only a few studies have been reported on the synergies between *Melissa officinalis* and other plants. Due to the volatile compounds in *Melissa officinalis* essential oil, the following effects have been reported: antifungal, antioxidant, antidiabetic, antibacterial and antimicrobial effects. These effects have also been reported in various extracts of *Melissa officinalis*.

In some research articles, in vitro and in vivo studies have been done to attribute pharmacological effects to *Melissa officinalis*, but more studies are needed in order to correlate the biological activity with the presence of certain components, and finally to be able to design specific compositions to obtain the desired biological activity.

## 4. Controlled Release Systems

Recent medical research has been related to the development of drug delivery systems containing natural or synthetic active substances (including those of herbal origin) with the main aim to provide biological active agents according to a proper kinetic, in order to protect them against harsh conditions or to assure targeted delivery [106]. The use of new drug delivery systems to transport drugs to certain parts of the body is an option that could solve various problems like in vivo instability, poor bioavailability, low solubility, and poor absorption in the body [107]. When a drug is introduced into the human body using traditional methods of administration, there are a lot of biotransformations that occur as a result of the drug’s interaction with the biological environment. Drug delivery systems have been designed to change the pharmacokinetics of drugs; thus, the major objectives of nanomedicine in terms of controlled drug administration are to maximize the bioavailability and efficacy of drugs, and to control their pharmacokinetics, pharmacodynamics, nonspecific toxicity, immunogenicity, and biorecognition [108,109].

A drug delivery system refers to a technical system that comprehensively regulates the availability of the drugs in living organisms in terms of the place of delivery, time and dose. The main objectives of a drug delivery system is to ensure the controlled release of drugs and their targeted delivery, to improve solubility and stability, to regulate metabolism and promote absorption, and to transport drugs across biological barriers [110]. These systems are designed to deliver the active content in a predictable in vivo pattern over an expected period of time [111]. 

The development of nanotechnology has led to the formation of specific nano-carriers of organic or inorganic, natural or synthetic origin. Nano-carriers are materials with nanometric dimensions which are used as vehicles for the transport of the desired biologically active agents [112]. The incorporation of plant extracts in these carriers (such as vesicles, microspheres, nanoemulsions, polymeric nanoparticles, nanocapsules, solid lipid nanoparticles, or phytosomes, etc.) has proven to be a new technology with a lot of advantages for the administration of herbal medicines [113,114].

Drugs typically interact specifically with biological receptors or molecules, such as enzymes, to produce physiological or pharmacological effects, either by activating or inhibiting these biological targets [115]. The main challenge is to develop an effective formulation that combines the active compounds of interest with a suitable delivery system to produce the desired effect [109].

**Figure 2:**
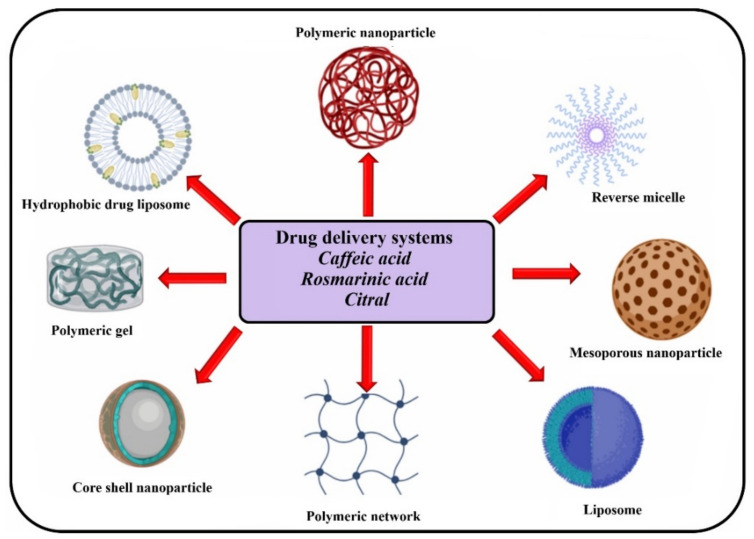
Drug delivery systems for *Melissa officinalis* components (realized with BioRender.com, accessed on 7 March 2022).

Among recent research studies, *Melissa officinalis* is one of the medicinal plants from which bioactive compounds are extracted in order to be introduced into controlled release systems (Figure 2). Furthermore, some of the components also found in *Melissa officinalis* extracts have been used in various research studies as active compounds incorporated into controlled release systems.

### 4.1. Drug Delivery Systems Based on Pure Components Which Are Available in Melissa officinalis

Depending on the type of delivery system and the encapsulated active substance, numerous controlled release systems with different biological effects have been developed (Table 6).

Nanoparticles used as delivery vehicles consist of various biodegradable materials, such as natural or synthetic polymers (including proteins, lipids, polysaccharides, lactides, polyurethanes, and polyglycols, etc.), ceramic powders, metals and metal oxides, and composites, etc. [131]. The composition and morphology of nanoparticles can be adapted to produce a suitable release profile in order to cross the biological barriers in the body [132]. 

Caffeic acid and rosmarinic acid are phenolic acids which are naturally present in many plants, and it is known that these phenolic compounds are antioxidants which can be used to fight against many diseases. In the study of Sguizzato et al. [118], it was proposed to load caffeic acid into solid lipid nanoparticles based on polaxamer. Caffeic acid-loaded solid lipid nanoparticles were produced by a method based on lipid fusion, homogenization and ultrasound treatment. The results of this study confirmed the production of a nanoparticulate gel with caffeic acid, following their previous study on the incorporation of caffeic acid into solid lipid nanoparticles [117]. In both studies, the encapsulation of caffeic acid in solid lipid nanoparticles has been shown to be promising in the application of caffeic acid to the skin, and/or for its delivery to the colon. Subsequently, in a study by Hallan et al. [116], a comparison was made between solid lipid nanoparticles and ethosomal vesicles regarding the skin delivery of caffeic acid in which the antioxidant activity and permeability of caffeic acid in the skin were evaluated in vitro, and an in vivo comparative irritation test was performed. 

Wani et al. [121] developed rosmarinic-acid-loaded chitosan nanoparticles, and their in vitro and in vivo release properties were evaluated. Chitosan is a natural polysaccharide with versatile biopolymeric properties. Due to its properties, chitosan is recognized for biomedical use as a safe material; it can be used in various applications, including drug delivery [133]. Rosmarinic acid is a polyphenolic compound with biological activity monitored in many studies with plant extracts. da Silva et al. [119,120] studied the encapsulation in chitosan nanoparticles of rosmarinic acid from *Salvia officinalis* and *Satureja montana* extracts. In a subsequent study, they showed that the obtained nanoparticles can be used as drug delivery systems for ocular applications in oxidative eye conditions. Chung et al. [122] synthesized nanoparticles from poly(ethylene glycol) (PEG) and rosmarinic acid, and found that this type of nanoparticle is a promising alternative in the treatment of various inflammatory diseases. The results obtained by Bhatt et al. [123] confirmed that the formulation of solid lipid nanoparticles as a drug delivery system loaded with rosmarinic acid is a non-invasive nose-to-brain drug delivery system, and that it is a promising approach for the treatment of Huntington’s disease.

Mesoporous silica nanoparticles are efficient delivery systems compared to other materials they have demonstrated excellent properties for applications in the biological system. Depending on the synthesis conditions, different types of mesoporous silica with variable pore sizes can be obtained.

Arriagada et al. [124] developed mesoporous silica nanoparticles loaded with rosmarinic acid and morin hydrate (a flavonoid found in fruits and plants). The results of the encapsulation of rosmarinic acid and morin hydrate in mesoporous silica indicated strong antioxidant effects. After studying the release kinetics of the substances, the authors of the article recommend the incorporation of antioxidant nanosystems into pharmaceutical formulations for the release of the active substances in the intestine.

Citral is the main component of the essential oil of the dried leaves of *Melissa officinalis*; Nordin et al. [125] loaded it into lipid nanoparticles for the administration of anti-cancer drugs. In this study, the nanoparticles obtained by the loading of citral in a nanostructured lipid carrrier system were characterized, and their safety profiles were examined in vitro and in vivo by observing that they are not toxic. Subsequently, Nordin et al. [126] reported that a citral-loaded nanostructured lipid carrier is an effective delivery system for triple-negative breast cancer treatments. 

Hydrogels are a class of materials with multiple properties, including a matrix for the encapsulation, transport and release of drugs. Hydrogels based on polysaccharide, especially those based on chitosan, provide a high degree of biocompatibility. Ailincai et al. [127,128] created a series of drug delivery systems prepared by the hydrogelation of chitosan with citral in the presence of an antineoplastic drug 5-fluorouracil; in the following study, they designed and characterized the hydrogels obtained by reacting PEGylated chitosan derivatives with citral. By following the in vitro and in vivo release kinetics, the efficacy of these drug delivery systems was established.

In a comparative study, Usach et al. [129] studied the loading of the essential oil from the leaves of *Citrus limon* var. *pompia* and citral into phospholipid vesicles. The phospholipid vesicles with essential oil were compared with the phospholipid vesicles containing citral, and it was found that they are small in size; both showed antimicrobial activity, but the vesicles with citral were slightly more effective against *Escherichia coli*, *Staphylococcus aureus* and *Candida albicans*.

β-caryophyllene is a volatile compound with many biological activities; it is found in large quantities in essential oil extracted from various plants. Because β-caryophyllene has a high level of lipophilicity and poor stability in hydrophilic environments, several complex systems have been tried. Di Sotto et al. [130] studied the preparation of liposomes from phosphatidylcholine in soy with β-caryophyllene by the thin-film hydration method, followed by extrusion. The results of the study showed that liposomes loaded with β-caryophyllene are potential effective delivery systems, and that the parameters of the lipid–drug ratio and lamerity are important for the release of β-caryophyllene by liposomes.

Scientific advances are based on research into biomaterials which are compatible with the biologically active constituents of extracts in order to revolutionize drug delivery systems. At present, there is a continuous demand for new biomaterials at the nano scale because the interest for the development of nanomedicine is very high [107].

### 4.2. Drug Delivery Systems Based on Melissa officinalis Extracts

Regarding the expansion of research into new controlled release systems containing active biocomponents extracted from natural plants, *Melissa officinalis* is one of the recently studied plants. Different types of *Melissa officinalis* extracts incorporated into matrices in the form of glycerosomes, films, hydrogels, dispersions and nanocapsules have been used to form controlled release systems (Table 7).

Antiherpetic activity has been demonstrated in two studies. Vanti et al. [134] studied the loading of *Melissa officinalis* essential oil inside glycerosomes, and they evaluated these vesicles for anti-herpetic activity against HSV type 1. Initially, they tried to obtain liposomal vesicles with phosphatidylcholine (P90G) and cholesterol in different ratios, but they were difficult to reproduce and poorly stable, so they approached the situation differently and made blisters out of a lipid film. The lipid film was composed of P90G and cholesterol, and was then hydrated under various conditions with a 10% *v*/*v* glycerol/water solution; it was then loaded into glycerosomes at 10 mg/mL essential oil of *Melissa officinalis* by the thin-layer evaporation method, in two steps. Analyses and tests showed that they obtained spherical glycosomes with several lamellae, which were nano-sized and able to pass through the pores of the skin, and were also able keep the constituents of *Melissa officinalis* essential oil from the degradation process during storage. In-vitro research showed that glycerosomes loaded with essential oil have a strong anti-HSV-1 activity, without cytotoxic effects.

In the second study, Rechia et al. [135] studied the properties of a drug administration system for the treatment of labial herpes. Four polymeric films with different concentrations of starch, glycerol and hydroalcoholic extract of *Melissa officinalis* were trialed. The results of the analysis showed that the increase in glycerol content in the films improved the characteristics of the drug delivery system, and that the films obtained from starch/glycerol/*Melissa officinalis* extract can be used for the treatment of labial herpes.

Due to the antimicrobial action of the essential oil, Serra et al. [138] found an antifungal drug to inhibit the growth of *Candida albicans* in the oral cavity. In this study, an attempt was made to encapsulate *Melissa officinalis* essential oil in methylcellulose hydrogels. Methylcellulose was used to prepare 10% (*w*/*v*) hydrogels with 1 and 2% (*w*/*v*) essential oil from *Melissa officinalis*. The analysis showed the use of hydrogels as drug delivery systems because methylcellulose hydrogel (10% (*w*/*v*)) with 2% (*v*/*v*) essential oil of *Melissa officinalis* significantly reduced the retention of *Candida albicans*.

Najafi-Soulari et al. [139] investigated the encapsulation of *Melissa officinalis* aqueous extract in calcium alginate hydrogel beads. Three significant parameters (extract, sodium alginate, and calcium chloride concentrations) were varied in order to obtain the maximum encapsulation efficiency. It was proven that the antioxidant activity of the extract did not change after encapsulation, and the most effective encapsulation was obtained with the concentration of sodium alginate solution, at 1.84%, the concentration of calcium chloride, at 0.2%, and the concentration of lemon balm extract, at 0.4%. In this study, they tried to cover the alginate beads with chitosan, but the results showed that the extract content is lost due to the immersion of the beads in the chitosan solution. However, the coated beads had better results for the release of the extract in the simulated intestinal fluid, indicating the protective effect of the chitosan layer.

Sani et al. [136,137,146] studied the encapsulation of *Melissa officinalis* essential oil in microcapsules and bioactive films. In the first study [146], the encapsulation of essential oil in isolated whey proteins and sodium caseinate was investigated using the ultrasonication technique. The results showed that ultrasound intensification had a negative impact, and that the smallest particle size was obtained at higher isolated whey protein levels, and with the lowest ultrasonic power. Sani et al. [136,137] produced bioactive films from chitosan and zinc oxide nanoparticles—respectively, sodium caseinate and zinc oxide nanoparticles—in which *Melissa officinalis* essential oil was encapsulated, and the antimicrobial properties of the obtained films were tested.

Santamaria-Echart et al. [140] studied different ways of incorporating extracts of *Melissa officinalis* and *Salvia officinalis* into water-based polyurethane–urea dispersions. The plant extracts were obtained by infusion, and were incorporated into water-based polyurethane-urea dispersions in different phases of the production process (the post-, in-situ and pre-methods). Regarding the antibacterial properties, the results showed that the properties of the two extracts differed depending on the method of their incorporation into the polyurethane nanoparticles.

In order to create a bioactive surface, Grumezescu et al. [142] functionalized magnetic microspheres with *Melissa officinalis* essential oil. The magnetic microspheres were composed of nanoparticles of magnetite (8 nm), essential oil, polylactic acid and chitosan using a solvent evaporation method, and were film-coated by MAPLE (Matrix Assisted Pulsed Laser Evaporation). In vitro experiments showed that the prepared surface had anti-staphylococcal properties.

Metallic nanoparticles are used in numerous research studies; as such, Fierascu et al. [29] synthesized silver, gold metallic nanoparticles, and gold–silver bimetallic nanoparticles with ethanolic extract from *Melissa officinalis*. The analytical results showed that silver nanoparticles with a diameter of about 13 nm, gold nanoparticles with a diameter of about 10 nm, and bimetallic nanoparticles composed of small particles of about 8 nm with a flower-like structure were obtained. The nanoparticles obtained were tested for their antimicrobial properties and mutagenicity, but it was observed that bimetallic nanoparticles have an intermediate biological action, and can be used further in applications.

In the study by Ahmeda et al. [143], *Melissa officinalis* aqueous extract incorporated into silver nanoparticles had a pharmaceutical potential similar to mitoxantrone (a chemotherapeutic agent). The nanoparticles obtained were spherical, measuring between 5–30 nm, and it was observed that the use of a higher concentration of extract decreases the average size of the silver nanoparticles. By performing in vitro and in vivo experiments, similar results were obtained with mitroxantrone for the treatment of acute myeloid leukemia, and the excellent antioxidant and cytotoxic properties of silver nanoparticles with *Melissa officinalis* extract were observed. Ruiz-Baltazar et al. [144,147] performed a study on the synthesis of silver nanoparticles, and in the next study they synthesized a silver-hydroxyapatite nanocomposite. In both studies, the extract of *Melissa officinalis* obtained by infusion was incorporated into the silver nanoparticles. It was observed that the functionalized nanoparticles showed a very good interaction with the hydroxyapatite matrix because its structure allows the substitution of calcium ions (Ca^2+^) with other metal ions such as silver (Ag^+^). The antibacterial properties of silver nanoparticles functionalized with *Melissa officinalis* extract were tested, but the silver–hydroxyapatite nanocomposite was characterized only chemically and structurally.

The results of the studies showed the compatibility of *Melissa officinalis* extracts with different types of carrier materials. Some systems could be enhanced with different substances in order to obtain materials with the best possible properties from systems in which different properties have been demonstrated, including increased stability, increased activity, increased solubility, and decreased toxicity [113].

The incorporation of zinc oxide nanoparticles and *Melissa officinalis* essential oil greatly improved the physical and mechanical properties of chitosan [136] and sodium caseinate films [137]. In the case of the systems obtained from starch, glycerol and *Melissa officinalis* extract, an increase of the characteristics of the films obtained was observed with the increase of the glycerol content [135].

There are many examples in the literature, such as those listed, but one needs to know the scope of the materials and the desired characteristics to be improved with different substances.

Controlled release systems offer many advantages in the development of medicine, but most of the findings focus on acute toxicity [148]. In order to evaluate the toxicity and safety of a system, long-term toxicity studies are required, as various interactions may occur between the active substance, the material, and the biological environment, in time. At the cellular level, various reactions may occur as a result of the encounter with the nanomaterial, such that various changes in the balance between inflammatory and anti-inflammatory mechanisms can be identified [149]. Toxicological analyzes are critical, and play a key role in the development of new systems [150]. Thus, in order to be officially approved as a medicinal product, controlled release systems must be subjected to in vitro and in vivo tests of their toxicology within the preclinical development phase.

## 5. Conclusions and Future Perspectives

This review summarized the phytochemical composition and pharmacological effects of *Melissa officinalis*, as well as the controlled release systems which have been investigated to date. Studies on the chemical composition of the essential oil and many types of extracts have been reported; depending on the area, the period, and the method of harvesting the plant, different concentrations of active substances have been obtained. The pharmacological effects of the extracts are mainly assigned to the presence of large amounts of polyphenolic compounds, such as antioxidant, antimicrobial, antiproliferative, and cytotoxic effects, and so on. By investigating the mechanisms of action and pharmacokinetics of the extracts and active compounds, new systems with biological activities for the human body and the environment can be obtained. The controlled release systems developed so far represent a future perspective for the development of new systems. These systems may contain other materials as a delivery system, or those made so far may be improved. With many substances, essential oils, and natural plant extracts available, many materials can be functionalized in order to develop controlled release systems. Materials such as silica, polysaccharides, polymers, and lipids can be used as encapsulation carriers. Functionalized controlled release systems can be enhanced with various substances so that they can be used, depending on the desired field. 

As a future perspective, the phytochemical composition and pharmacological effects attributed to *Melissa officinalis* represent an opportunity to create new controlled release systems with the potential for targeted delivery.

## Figures and Tables

**Table 1:** Components of the essential oil extracted from the dried leaves of *Melissa officinalis*.

Component Name	Concentration of the Components of the Essential Oil, %	Reference
Majority components		
(E)-Caryophyllene	1.06–6.8	[12,13,14]
Caryophyllene oxide	1.3–43.55	[12,13,14,15,16]
Citronellal	0.4–20.3	[12,13,15,16,17]
Geranial (citral A)	6.22–51.21	[14,15,16,17,18]
Geranyl acetate	0.5–19.3	[12,13,14,17]
Neral (citral B)	4.28–35.02	[12,13,14,15,16,17,18]
α-Cadinol	5.64	[14]
α-Copaene	0.1–7.02	[12,15,16]
β-Caryophyllene	1.3–29.14	[14,15,16,17]
Minority components (<5%)		
(2E)-Nonen-1-al	0.2	[12]
(E)-Nerolidol	0.2	[12]
(E)-α-Bergamotene	1.24	[14]
(E)-β-Ionone	0.9	[12]
(E)-β-Ocimene	0.1–0.5	[12,13]
(E-E)-Geranyl linalool	1.59	[14]
(Z)-β-Ocimene	0.1	[15]
1,2-Benzenedicarboxilic acid, butyl 2-methylopropyl ester	0.6	[13]
1,8-Dehydro-cineol	0.1	[13]
14-Hydroxy-9-epi-(E)-caryophyllene	0.2	[13]
1-Octen-3-ol	0.2–0.3	[12,13,15]
3,5-Heptadienal,2-ethylidene-6-methyl	0.4	[13]
3-Octanone	0.2	[17]
6-Methyl-5-hepten-2-ol	0.2–1.7	[12,13,15]
Benzene acetaldehyde	0.3	[12]
Camphene	0.38–1.38	[14,16]
Camphor	0.1–0.4	[13,15]
Carvacrol	0.3–1	[12,13]
Caryophyllenol	0.5–2.23	[14]
cis-2H-3a-Methyl-octahydro-Inden-2-one	4.7	[17]
Cis-Chrysanthenol	0.7–1.7	[12,13,15]
Cis-Rose oxide	0.1–0.2	[12,15]
Citronellol	0.4–1.88	[12,14]
Citronellyl acetate	0.1	[12]
Dihydrocitronellol acetate	0.3	[15]
Geraniol	0.6–0.7	[12,13,15]
Germacrene D	0.2–2.0	[12,13,14]
Humulene epoxide II	0.2–1.29	[13,14]
iso Aromadendren epoxide	0.46	[14]
Isogeranial	1.4–2.0	[13]
Isomenthol	2.4	[15]
Linalool	0.3–0.5	[12,15]
Linalool + trans-Sabinene hydrate	0.5–0.8	[13]
Menthol	0.3	[15]
Methyl citronellate	0.5–2.78	[12,13,16]
Methyl eugenol	0.1	[12]
Methyl geranate	0.2–0.4	[12,13,17]
Myrcene	0.1–0.3	[13,15]
n-Eicosane	0.6	[15]
Nerol	0.2	[15]
Neryl acetate	0.1	[12]
n-Heneicosane	0.4	[15]
n-Nonanal	0.1–0.4	[12,15]
para-Mentha-1(7),8-diene	0.1	[13]
p-Cymene	0.1	[12,13]
Phytol	3.64	[14]
Rosefuran epoxide	0.6–0.7	[13]
Sabinene	0.4	[13]
Thymol	0.1–3.1	[12,13,14]
t-Muurolol	0.59	[14]
trans-Limonene oxide	0.6	[13]
trans-para-Mentha-1(7),8-dien-2-ol	2.3	[17]
Trans-Rose oxide	0.1	[12,15]
Valencene	0.1	[15]
α-Humulene	0.2–2.6	[12,13,15,16]
α-Calacorene	0.76	[14]
α-Cubebene	0.42–1.23	[14]
β-Cubebene	0.1	[15]
β-Pinene oxide	1.1	[13]
β-sesquiphellandrene	0.97	[14]
γ-Cadinene	0.76–1.77	[14]
γ-Terpinene	0.3–0.5	[12,13]

Traces of components were not taken into account (contents below 0.05%).

**Table 2:** Triterpenes from *Melissa officinalis* extracts.

Component Name	Content *, μg/g	Part of Plant	Reference
Betulinic acid	12.85–169.88	aerial parts	[4]
Oleanolic acid	915.03–6151.67	aerial parts	[4]
Ursolic acid	3577.00–11,234.97	aerial parts	[4]
23-Sulfate ester of niga-ichigoside F1	n.a.	leaves and stems	[25]
3β,16β,23-Trihydroxy-13,28-epoxyurs-11-ene-3-O-β-D-glucopyranoside	n.a.	dried leaves and stems	[24]
3,23-Disulfate ester of 2α,3β,19α,23-tetrahydroxyurs-12-en-28-oicacid	n.a.	dried leaves and stems	[24]
3,23-Disulfate ester of 2α,3β,19α,23-tetrahydroxyurs-12-en-28-oicacid 28-O-β-D-glucopyranoside	n.a.	dried leaves and stems	[24]
3,23-Disulfate ester of2α,3β,23,29-tetrahydroxyolean-12-en-28-oicacid	n.a.	dried leaves and stems	[24]
3,23-disulfate ester of 3β-23,29-trihydroxyolean-12-en-28-oic acid	n.a.	dried leaves and stems	[24]
3,23-Disulfate ester of 2α,3β-23,29-tetrahydroxyolean-12-ene-28-oicacid	n.a.	dried leaves and stems	[24]
23-sulfate ester of 2α,3β,19 α,23-tetrahydroxyurs-12-en-28-oic acid	n.a.	fresh leaves and stems	[26]
23-sulfate ester of 2α,3β,19 α,23-tetrahydroxyurs-12-en-28-oic acid 28-O-β- D-glucopyranoside	n.a.	fresh leaves and stems	[26]
Melissioside A	n.a.	leaves and stems	[25]
Melissioside B	n.a.	leaves and stems	[25]
Melissioside C	n.a.	leaves and stems	[25]

n.a. = not available; * expressed on a dry weight basis.

**Table 3:** Major polyphenolic compounds from *Melissa officinalis* extracts.

Group Name	Compound Name	Content *, μg/g	Part of Plant	Reference
Phenolic acids	Caffeic acid	39.38–860.72	Dried leaves	[4]
	Caftaric acid	1.85–344.34	Dried leaves	[4]
	Chlorogenic acid	0.62–75.529	Dried leaves	[4,29]
	Ferulic acid	1.03–45.489	Dried leaves	[4,29]
	Gentisic acid	10.40–60.48	Dried leaves	[4]
	p-Coumaric acid	1.06–20.72	Dried leaves	[4]
		13.37 ± 2.84	Aerialparts	[30]
	Rosmarinic acid	3515.60–86,637.60	Dried leaves	[4]
		6914.1 ± 779	Aerial parts	[30]
Flavonoids	Apigenin	0.66–84.53	Dried leaves	[4,29]
		41.71 ± 20.6	Aerial parts	[30]
	Cynaroside	408.13 ± 30.0	Aerial parts	[30]
	Daidzein	51.25 ± 8.07	Aerial parts	[30]
	Hyperoside	3.30–16.240	Dried leaves	[4]
	Isoquercetin	6.82–162.40	Dried leaves	[4]
	Kaempherol	21.84	Dried leaves	[4]
	Luteolin	0.81–26.32	Dried leaves	[4]
	Myricetin	3.45–17.92	Dried leaves	[4]
	Quercetin	153.46	Dried leaves	[29]
	Quercetrol	5.72–33.60	Dried leaves	[4]
	Rutin	8.11–1462.99	Dried leaves	[4,29]

* expressed on a dry weight basis.

**Table 4:** Pharmacological effects reported from *Melissa officinalis* extracts.

Effect	Model	Dosage or Concentration	Tested Systems	Results	Type of Extract	Reference
Antiproliferative	in vitro	20, 100, 250 μg/mL	Breast cancer cells MDA-MB- 231 and healthy HaCat cells	Inhibitory effect on migration and proliferation of both types of cells	ethanolic extract	[38]
	in vitro	50%	Human Colon Cancer Cell Line (HCT-116)	The 50 % ethanolic extract showed significant differences after 72 h of treatment, reducing cell proliferation to values close to 40%	ethanolic and aqueous extracts	[39]
Antitumor	in vitro	Different concentration	Human tumor cell lines: MCF-7, AGS and NCI-H460	Obtained revealed that the ethanolic extract presented the highest cell growth inhibitory potential in all the human tumor cell lines tested	ethanolic, methanolic, hydro-methanolic, hydro-ethanolic and aqueous extracts)	[40]
Antioxidant	in vitro	Different concentration	Encephalic tissue from male Wistar rats	Effective agent in the prevention of various neurological diseases associated with oxidative stress	ethanolic, methanolic and aqueous extracts	[41]
	in vitro	1, 2.5, 5 and 10 mg/mL	DPPH radical scavenging activity assay, β-carotene bleaching test and ABTS assay	Good antioxidant activity	essential oil	[42]
Antiangiogenic	in vitro, in ovo	50 μg/mL	Two breast cancer cell lines, MCF-7 And MDA-MB-231	Highest cell inhibitory activity was exhibited by the 96% ethanolic extract	ethanolic extracts and methanolic extracts	[4]
Cardioprotective	in vivo	25, 50 and 100 mg/kg b.w. * (4.23/8.46/16.91 mg/kg b.w. *)	Rats	Antioxidant and cardio-protective effects against arrhythmias induced by ischemia and ischemia-reperfusion	ethanolic leaf extract	[43]
Antinociceptive Antihyperglycemic	in vivo	0.01, 0.02 and 0.04 mg/day (0.0063/0.0126/0.0252 mg/kg b.w. *)	Male adult Wistar rats	Long-term oral administration of essential oil (at an effective dose of 0.04 mg/day) can suppress chemical hyperalgesia in diabetic rats	essential oil	[44]
Anxiolytic Antidepressant	in vivo	50, 75 and 150 mg/kg b.w./day * (3.91/5.86/11.72 mg/kg b.w. *)	Albino BALB/c male mice	Hydro-alcoholic extract (75 and 150 mg/kg) significantly reversed anxiety- and depressive-like behaviors	hydro-alcoholic extract	[45]
Neuroprotective	in vitro	5, 10, 50, 100, 500 μg/mL	Cortical neuronal Culture system	Protective effects on neurons in the brain	balm oil	[46]
	in vivo	50, 100, 200 and 400 mg/kg b.w. * (8.35/16.71/33.41/66.83 mg/kg b.w. *)	Male rats	Treatment with 100 mg/kg of oil attenuated the increased caspase-3 like protease activity significantly	balm oil	[46]
GABA-T inhibitor	in vitro	0–4 mg/mL	Rat brain	Phytochemical characterization of the crude extract determined rosmarinic acid as the major compound responsible for activity (40% inhibition at 100 μg/mL) since it represented approximately 1.5% of the dry mass of the leaves	methanol extract	[47]
Anti-kinetoplastidae	in vitro	31.25, 62.5, 125, 250 μg/mL	*T. cruzi*, *L. brasiliensi*, *L. infantum*	A potential source of natural product featuring anti-*Leishmania* and anti-*Trypanosoma* activity	ethanol extract	[48]
Analgesic	in vivo	5, 10, 20 mg/kg b.w. * (0.87/1.73/3.46 mg/kg b.w. *)	Male Wistar rats	Intrathecal administration could significantly improve hot-water and formalin-induced pain in male Wistar rats	hydro-alcoholic extract	[49]
Hypnotic	in vivo	200, 400 and 800 mg/kg b.w. * (14.81/29.61/59.23 mg/kg b.w. *)	Male Swiss mice	Extracts may be useful for insomnia	hydro-alcoholic extracts	[50]
Antidiabetic	in vivo	0.0125 mg/d	db/db mice	Anti-hyperglycaemic agent	essential oil	[51]
	in vivo	0.4%, 0.8% (*w*/*w*)	Otsuka Long-Evans Tokushima fatty rats	An effective therapeutic strategy to treat human obesity and type 2 diabetes	herbal extract	[52]
Anti-Alzheimer	in vitro	8.8 mg/mL	GSK-3Β, CK-1δ, and BACE-1	Best activity for ck-1δ inhibitory activity with maximum inhibitory concentration values at half (IC50) below 250 μg/mL	methanol extract	[53]
Antispasmodic	ex vivo	1, 5, 10, 25, and 50 mg/mL	Different segments of the gastrointestinal tract of mice	Site- and dose-dependent effects on the contractile activity of the gastrointestinal tract	hydro-ethanolic leaf extract	[54]
Antiviral	in vitro	1.5–150 μg/mL	RC-37 cells	High virucidal activity against HSV-1	aqueous extract	[55]
Antifungal	in vitro	15.5–2000 μg/mL	Human Pathogenic fungi	Good antifungal activity	ethanol extracts	[56]
		0.25–2 μL/mL	Phytopathogenic fungi in apples		essential oil	[57]
Antibacterial	in vitro	10 and 15 mg/mL	*E. coli*, *L. monocytogenes*, *S. aureus* and *S. typhimurium*	A significant antimicrobial effect	essential oil	[42]

* estimated human equivalent dose. b.w. = body weight.

**Table 5:** Main components of *Melissa officinalis* and their activity.

Substance	Activity	Reference
Geranial (citral A)	Antibacterial, antifungal	[69,70]
Neral (citral B)	Antibacterial, antifungal	[71,72]
Citronellal	Antimicrobial, insecticidal	[73,74]
β-Caryophyllene	Anti-inflammatory, antioxidant, antibacterial	[75,76]
α-Cadinol	Antifungal, hepatoprotective	[77,78]
Geranyl acetate	Antibacterial, insecticidal	[79,80]
Betulinic acid	Antiviral, anti-inflammatory, anticancer	[81,82]
Oleanolic acid	Antiviral, hepatoprotective	[83,84]
Ursolic acid	Antibacterial, antioxidant	[85,86]
Caffeic acid	Antioxidant, anti-inflammatory	[87,88]
Caftaric acid	Antioxidant	[89,90]
Rosmarinic acid	Antioxidant, anti-inflammatory	[91,92]
Ferulic acid	Antioxidant	[93,94]
Chlorogenic acid	Antidiabetic, antioxidant	[95,96]
p-Coumaric acid	Antioxidant, anti-inflammatory	[97,98]
Cynaroside	Antioxidant, anti-inflammatory	[99,100]
Rutin	Antioxidant, anti-inflammatory	[101,102]

**Table 6:** Controlled release systems with active substances which are available in *Melissa officinalis*.

Type of Delivery System	Delivery System		Effect	Reference
	Type of Carrier	Active Agent		
Organic/Inorganic Nanoparticles	poloxamer, soybean lecithin ethosome	caffeic acid	antioxidant	[116]
	chitosan, sodium alginate			[117]
	poloxamer			[118]
	chitosan	rosmarinic acid *	antioxidant	[119,120]
		rosmarinic acid	antimicrobial	[121]
	PEG-containing amine		anti-inflammatory	[122]
	glycerol monostearate, soya lecithin, hydrogenated soya phosphatidyl choline		therapeutic	[123]
	mesoporous silica		antioxidant	[124]
	nanostructured lipid	citral	anticancer	[125,126]
Hydrogels	chitosan	citral	anticancer	[127]
	imine-PEG-ylated chitosan		local therapy	[128]
Vesicles	soybean phospholipids	citral	antimicrobial	[129]
	ethosome	caffeic acid	antioxidant	[116]
	soybean phosphatidyl-choline liposomes	β-caryophyllene	antiproliferative	[130]

* from a natural extract.

**Table 7:** Controlled release systems with *Melissa officinalis* extracts.

Type of Delivery System	Delivery System		Effect	Reference
	Type of Carrier	Type of Extract		
Glycerosomes	phosphatidylcholine and cholesterol	essential oil	anti-herpetic	[134]
Films	starch and glycerol	Hydroalcoholic extract	anti-herpetic	[135]
	chitosan and zinc oxide nanoparticles	essential oil	antimicrobial	[136,137]
	sodium caseinate and zinc oxide nanoparticles			
Hydrogel	methylcellulose	essential oil	antimicrobial for candida albicans in the oral cavity	[138]
	calcium alginate	aqueous extract	antioxidant	[139]
Dispersion	water-based polyurethane-urea	infusion	antimicrobial	[140]
Nanocapsule	isolated whey proteins and sodium caseinate	essential oil	n.a.	[141]
	nanoparticles of magnetite, essential oil, polylactic acid, chitosan	essential oil	anti-staphylococcal	[142]
Nanoparticle	silver, gold and gold-silver	ethanolic extract	antimicrobial	[29]
	silver	aqueous extract	antioxidant and cytotoxic for the acute myeloid leukemia	[143]
	silver	infusion	antibacterial	[144]
Nanocomposite	silver-hydroxyapatite	infusion	antibacterial	[145]

n.a. = not available.

## Data Availability

Available on demand.
